# First study on the immunohistochemical expression of cyclooxygenase-2 and clinicopathological association in canine hepatoid gland neoplasms

**DOI:** 10.14202/vetworld.2022.2432-2441

**Published:** 2022-10-20

**Authors:** Pinkarn Chantawong, Thanongsak Mamom, Sahatchai Tangtrongsup, Setthakit Chitsanoor, Hassadin Boonsriroj

**Affiliations:** 1 Department of Companion Animal and Wildlife Clinic, Faculty of Veterinary Medicine, Chiang Mai University, Chiang Mai 50100, Thailand; 2 Integrative Research Center for Veterinary Preventive Medicine, Faculty of Veterinary Medicine, Chiang Mai University, Chiang Mai 50100, Thailand; 3 Department of Veterinary Pathology, Faculty of Veterinary Medicine, Mahanakorn University of Technology, Bangkok 10530, Thailand; 4 Mahanakorn Veterinary Diagnostic Center, Faculty of Veterinary Medicine, Mahanakorn University of Technology, Bangkok 10530, Thailand; 5 Research Center of Producing and Development of Products and Innovations for Animal Health and Production, Chiang Mai University, Chiang Mai 50100, Thailand

**Keywords:** canine, clinicopathological features, cyclooxygenase-2, hepatoid gland neoplasias, immunohistochemistry

## Abstract

**Background and Aim:**

Hepatoid gland neoplasms (HGNs) constitute one of the most common cutaneous tumors that arise from perianal glands in dogs and are clinically characterized by rapid growth. Cyclooxygenase-2 (COX-2), the inducible form of the enzyme, is associated with several hallmarks of tumorigenesis. Its expression has been confirmed in several human and animal neoplastic tissues, but there are no reports in hepatoid gland tissues. Therefore, this study aimed to investigate COX-2 immunoexpression in canine HGNs, compare the expression among groups of normal hepatoid glands, hepatoid gland adenomas (HGAs), hepatoid gland epitheliomas (HGEs), and hepatoid gland carcinomas (HGCs), and assess the association of the COX-2 expression with clinicopathological features.

**Materials and Methods:**

Sixty-one formalin-fixed paraffin-embedded canine hepatoid gland tissues (20 samples of HGAs, 16 of HGEs, 15 of HGCs, and 10 of normal hepatoid glands) were analyzed for COX-2 expression using immunohistochemistry with scoring for percentage positivity and intensity. Multiple comparisons of COX-2 expression among normal and neoplastic hepatoid glands and the associations between COX-2 expression and clinicopathological features were analyzed.

**Results:**

Cyclooxygenase-2 expression was not detected in 60% of normal hepatoid glands and 25% of HGAs. Seventy-five percent of HGAs had a weak expression, while 43.7% and 56.3% of HGEs showed weak and moderate expression, respectively. The expression of HGCs ranged from weak (13.3%) to moderate (33.3%) and strong (53.3%). The immunoreactivity score of COX-2 labeling was significantly different among the normal and neoplastic hepatoid glands (p < 0.0001). The highest score was observed in the HGCs. Only in HGCs, the strong COX-2 expression was significantly associated with some clinicopathological features, including tissue invasion (p = 0.007) and necrosis (p = 0.029).

**Conclusion:**

These results suggest that COX-2 may play a role in the modulation of neoplastic cell growth. These preliminary data lead to further investigation on the potential of COX-2 expression as a prognostic indicator and COX-2 inhibitors for canine HGCs treatment.

## Introduction

Hepatoid gland neoplasms (HGNs) are common skin tumors in dogs, accounting for 25% of all cutaneous epithelial tumors [1]. Their clinical characteristics are typically single or multiple masses surrounding the anus, tail, hind limbs, parapreputial, and vulva areas. Occasionally, they may induce an inflammatory response or surface ulceration [2]. Proliferative lesions of the hepatoid gland include adenomas, epitheliomas, and carcinomas. The benign hepatoid gland adenomas (HGAs) represent nearly 60% of all perianal tumors, and the prevalence has been reported at 13.04% of canine epithelial and skin gland tumors [3, 4]. The progressions of HGAs appear to be sex hormone-dependent [2]. Hepatoid gland epitheliomas (HGEs) are low-grade malignant, exhibit local invasiveness, and are the second most prevalent neoplasm of the perianal glands reaching 30% of cases [4]. Hepatoid gland carcinomas (HGCs) are the most aggressive type of HGNs due to their invasive and metastatic features and are responsible for 3–21% of all tumors in the perianal region [2]. Metastases occur in approximately 15–22% of cases with reports of spread to lymph nodes, abdominal organs, and lungs, causing severe systemic illness [5, 6]. Therapeutic managements, including surgical incision followed by chemotherapy with or without radiation, are highly recommended for HGCs [7]. However, non-resectable tumors with distant metastasis are not highly radiation responsive and effective treatment is needed to be developed [5].

Various inflammatory mediators are involved in the pathological process associated with cancer, such as prostaglandins (PGs). The production of PGs is controlled by the coordinated activity of eicosanoid-forming enzymes named Cyclooxygenase (COX) [8]. Two main isoforms of the enzyme have been identified as COX-1 and COX-2 [9]. The constitutively expressed COX-1 provides immediate mediators involved in homeostasis. In contrast, the inducible COX-2 is responsible for the production of PGE_2_, and it has been found to be upregulated in inflammatory and neoplastic conditions [10, 11]. The previous studies demonstrated that COX-2/PGE_2_ signaling pathways contributed to promoting the processes of malignant transformation and tumor progression by affecting cell proliferation, cell adhesion, angiogenesis, tissue invasion and metastasis, cell apoptosis, and immune-surveillance [12, 13]. The role of COX-2 in the progression of various cancers has become an area of extensive research. Cyclooxygenase-2 overexpression has been reported in various human cancers and is associated with poor prognosis in colon carcinoma, pulmonary carcinoma, breast carcinoma, malignant mesothelioma, squamous cell carcinoma, and chronic myelogenous leukemia [14–18]. In the veterinary literature, COX-2 overexpression has been documented in canine mammary carcinoma, cutaneous hemangiosarcoma, squamous cell carcinoma, renal cell carcinoma, mast cell tumors, cutaneous, and oral melanomas [19–24]. Recently, overexpression of COX-2 has been indicated in specific types of canine mammary carcinomas, including tubulopapillary, anaplastic, squamous cell carcinoma as well as carcinosarcomas [25, 26]. In addition, these expressions were strongly associated with the development of distant metastasis, a worse prognosis, and a shorter overall survival time [19, 27]. A relationship between COX-2 expression and tumor behavior prompted the investigation of using COX-2 inhibitors to control oncogenesis. Numerous studies have emphasized the efficacy of selective COX-2 inhibitors as chemoprevention and/or chemotherapy in human cancers such as colon, breast, bladder, and prostate cancers [28–31]. Epidemiological studies of patients receiving long-term COX-2 inhibitors indicated a significant decrease in the incidence and the multiplicity of colorectal carcinoma by inhibiting tumor angiogenesis, inducing tumor apoptosis, and preventing hepatic metastasis [32]. In animals, therapeutic studies have shown promising results in mammary, oral, and bone cancers [33–35]. Dogs with transitional cell carcinoma of the bladder and oral squamous cell carcinoma treated with piroxicam, a non-selective COX-2 inhibitor, exhibited the same rate of response to the treatment and survival time as those treated with traditional chemotherapy [33, 36]. Antineoplastic effects of COX-2 inhibitors include inhibition of cell cycle arrest, angiogenesis, and increasing the immune response at the tumor site [28, 37]. Therefore, COX-2 inhibitors might be an attractive alternative treatment for both human and animal neoplasia.

To the best of our knowledge, no studies have evaluated COX-2 expression in canine hepatoid gland tissues. The aims of this study were (1) to investigate COX-2 expression in canine HGNs, (2) to compare COX-2 expression among groups of normal hepatoid glands, HGAs, HGEs, and HGCs using immunohistochemistry with scoring for percentage positivity and intensity, and (3) to determine the association of COX-2 expression and clinicopathological features. This preliminary study could lead to further investigation of COX-2 targeted therapy in canine HGNs.

## Materials and Methods

### Ethical approval

The research protocols were approved by the Animal Ethics Committee of the Faculty of Veterinary Medicine, Chiang Mai University (Ref. No. R14/2563), under the guidelines for the Care and Use of Experimental Animals, National Research Council of Thailand.

### Study period and location

The study was conducted from December 2021 to March 2022 at the Veterinary Diagnostic Center, Faculty of Veterinary Medicine, Chiang Mai University, Chiang Mai, Thailand.

### Sample collection and histopathological examination

Fifty-one formalin-fixed paraffin-embedded (FFPE) samples of canine HGNs from January 2019 to December 2021 were retrieved from archives of Small Animal Veterinary Teaching Hospital, Faculty of Veterinary Medicine, Chiang Mai University, Chiang Mai and the Vet and Vitro Central Laboratory, Bangkok, Thailand. The individual data of dogs, including age, sex, history of neutering or spaying, breed, tumor size and location, skin ulceration and necrosis, tissue invasion, and lymphatic metastasis were recorded and defined as clinicopathological features. Normal hepatoid gland tissues were obtained postmortem from ten dogs (aged between 6 and 12 years old) from Veterinary Diagnostic Center, Faculty of Veterinary Medicine, Chiang Mai University.

To confirm the primary diagnosis, each FFPE tissue sample was cut into 4 mm thick, deparaffinized, and stained with hematoxylin and eosin. All samples were re-examined independently by three veterinary pathologists certified by the College of Veterinary Specialties of Thailand, according to the World Health Organization Classification of Tumors of Domestic Animals [38].

### Cyclooxygenase-2 immunohistochemistry

Immunohistochemical staining was performed using a polymer-based detection system (Novolink™ Polymer Detection System; Leica, Wetzlar, Germany), according to the manufacturer’s instructions. All FFPE samples were cut into 4 mm thick using an automatic microtome. The paraffin sections were routinely deparaffinized and rehydrated in xylene and graded ethanol, respectively. Then, antigen retrieval was performed using Epitope Retrieval Solution, pH 6.0 (Novocastra, Leica, Newcastle Upon Tyne, UK), by heating in a 750W microwave for 10 min. To block endogenous peroxidase activity, sections were treated with Novolink 3% H_2_O_2_ in methanol for 10 min. Nonspecific reactions were blocked by treating with Novolink 0.4% Casein in phosphate-buffered saline (PBS, pH 7.2) for 30 min. The tissue sections were incubated with 1: 100 primary antibody (rabbit anti-COX-2 polyclonal antibody; GeneTex, Irvine, CA, USA) at 4°C overnight in a humidified chamber. Sections then underwent Novolink post-primary block (rabbit anti-mouse immunoglobulin [Ig]G) for 30 min. After that, Novolink Polymer anti-rabbit Poly-horseradish peroxidase-IgG was applied and incubated at room temperature for 20 min. Visualization was performed using freshly prepared Novolink 3,3′-diaminobenzidine tetrahydrochloride solution for 10 min. The slides were counterstained with hematoxylin and mounted with coverslips using a permanent mounting medium. The canine mammary adenocarcinoma was used as positive and negative control [19]. For negative controls, the primary antibody was replaced with PBS.

### Immunohistochemical evaluation

COX-2 expression was indicated by the presence of brown cytoplasmic labeling. The immunohistochemical score (IHS) was determined by multiplying an estimate of the percentage of immunoreactive cells (quantity score) with an estimate of the staining intensity (intensity score) [14, 21]. The staining quantity was scored as follows: no cell staining scored as 0, 1–10% of cells as 1, 11–50% as 2, 51–80% as 3, and 81–100% as 4. The intensity of COX-2 immunoreactivity was graded as: 0 = no staining, 1= weak, 2 = moderate, and 3 = strong staining. The IHS of 9–12 was considered strong immunoreactivity, 5–8 as moderate, 1–4 as weak and 0 as negative.

### Statistical analysis

Descriptive statistics were used to describe the characteristics of each canine HGN type, including age, sex, history of neutering or spaying, breed, tumor size and location, skin ulceration, necrosis, tissue invasion, and lymphatic metastasis. The differences of COX-2 IHS among normal hepatoid glands and HGNs were analyzed using the Kruskal–Wallis test and *post hoc* multiple comparisons using Dunn’s test with Bonferroni adjustment. Fisher’s exact test was used to analyze (1) the association between clinicopathological features and type of HGNs and (2) the association of clinicopathological features with COX-2 expression in HGNs. All statistical analyses were performed using the STATA statistical software release 16.1 (STATA Corp., College Station, TX, USA). p < 0.05 was considered statistically significant.

## Results

A total of 51 HGNs tissue samples were obtained from dogs of different breeds and sexes (Tables-1 and 2). The mean age was 9.67 ± 2.67 years (range: 4–15 years). The majority of HGNs were located in the perianal area (n = 41, 80.4%). Mean diameter of the tumor was 3.90 ± 2.04 cm (range: 1.5–10 cm). Seven of 51 cases (13.7%) had evidence of lymphatic metastasis (Table-2). Normal hepatoid gland tissues were obtained postmortem from 10 dogs (intact males: n = 2; castrated males: n = 2; intact females: n = 2; and spayed females: n = 4) of different breeds (Table-1). The mean age was 8.2 ± 2.2 years (range: 6–12 years).

**Table-1:** Normal and neoplastic hepatoid gland samples from different dog breeds.

Dog breeds	Normal hepatoid glands	Neoplastic hepatoid glands
Mixed-breed	4	14
Shih Tzu	1	9
Poodle	3	6
Beagle	1	3
Golden Retriever	1	3
Siberian husky		6
Labrador Retriever		2
Miniature pincher		2
Chi Hua Hua		2
Jack Russel		1
Dachshund,		1
Thai-ridge back		1
Bang Kaew		1

**Table-2:** The association between clinicopathological features and type of canine HGNs.

Clinicopathologic features	Number of samples	Histology diagnosis			p-value

		HGA (n = 20) (%)	HGE (n = 16) (%)	HGC (n = 15) (%)	
Age (years)					0.086
<10	26	14 (53.9)	7 (26.9)	5 (19.2)	
>10	25	6 (24.0)	9 (36.0)	10 (40.0)	
Sex					0.009
Intact females	2	1 (50.0)	1 (50.0)	0 (0.0)	
Spayed females	6	2 (33.3)	1 (16.7)	3 (50.0)	
Castrated males	11	0 (0.0)	5 (45.5)	6 (54.5)	
Intact males	32	17 (53.1)	9 (28.1)	6 (18.8)	
Tumor size, cm					<0.001
<3	17	12 (70.6)	5 (29.4)	0 (0.0)	
3–5	24	8 (33.3)	10 (41.7)	6 (25.0)	
>5	10	0 (0.0)	1 (10.0)	9 (90.0)	
Location					0.217
Perianal	41	17 (41.4)	15 (36.6)	9 (22.0)	
Tail based	7	2 (28.6)	1 (14.3)	4 (57.1)	
Prepuce	2	1 (50.0)	0 (0.0)	1 (50.0)	
Perivulva	1	0 (0.0)	0 (0.0)	1 (100.0)	
Ulceration					0.038
Present	24	5 (20.8)	9 (37.5)	10 (41.7)	
Absent	27	15 (55.6)	7 (25.9)	5 (18.5)	
Necrosis					<0.001
Present	21	2 (9.5)	7 (33.3)	12 (57.1)	
Absent	30	18 (60.0)	9 (30.0)	3 (10.0)	
Tissue invasion					<0.001
Present	10	0 (0.0)	0 (0.0)	10 (100.0)	
Absent	41	20 (48.8)	16 (39.0)	5 (12.2)	
Lymphatic metastasis					0.001
Present	7	0 (0.0)	0 (0.0)	7 (100.0)	
Absent	37	17 (46.0)	13 (35.1)	7 (18.9)	
N/A	7	3 (42.9)	3 (42.9)	1 (14.3)	

HGA=Hepatoid gland adenomas, HGE=Hepatoid gland epithelioma, HGC=Hepatoid gland carcinoma, HGNs=Hepatoid gland neoplasms

**Figure-1:**
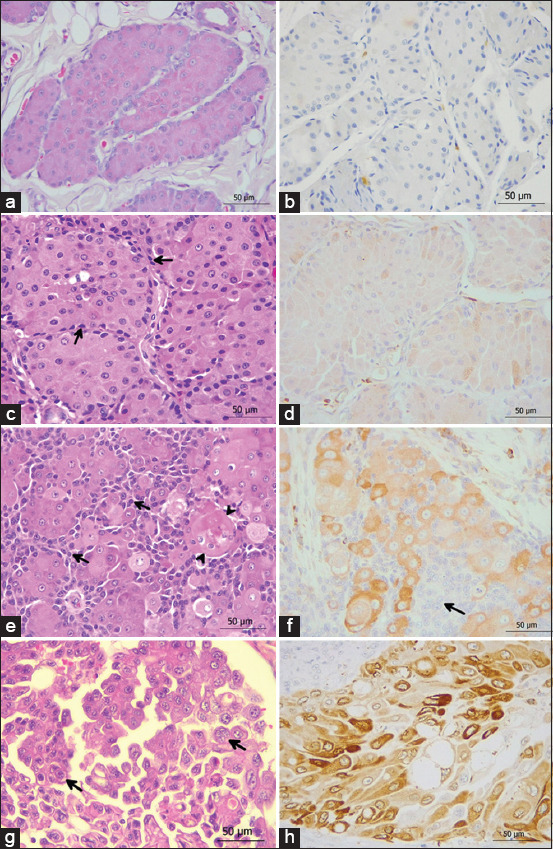
Histopathological and immunohistochemical features of normal canine hepatoid gland and hepatoid gland neoplasms. (a) Normal hepatoid gland; the glands composed of uniform epithelial cells with abundant eosinophilic cytoplasm arranged in trabeculae which separated by variable thickness of fibrovascular stroma. (b) The mature hepatoid cells and basaloid reserve cells were immunonegative for cyclooxygenase-2 (COX-2). (c) Hepatoid gland adenoma; well-differentiated hepatoid cells arranged in islands and trabeculae rimmed by basaloid reserve cells (arrows) and embedded in thin fibrovascular stroma. (d) Hepatoid cells were weak and sparse cytoplasmic immunoexpression of COX-2. (e) Hepatoid gland epithelioma; hepatoid cells with abundant eosinophilic cytoplasm organized in nests with thick reserve cells layers (arrows), and focal squamous metaplasia was present (arrowheads). (f) Hepatoid cells presented weak to moderate cytoplasmic immunoexpression, whereas basaloid reserve cells were immunonegative (arrow). (g) Hepatoid gland carcinoma with anaplastic features; pleomorphic neoplastic cells exhibited bizarre nuclei with moderate amount of eosinophilic cytoplasm (arrows). (h) Anaplastic cells showed strong COX-2 cytoplasmic immunoexpression. (a, c, e, g) Hematoxylin and Eosin, (400×). (b, d, f, h) Immunohistochemistry, (400×).

**Figure-2:**
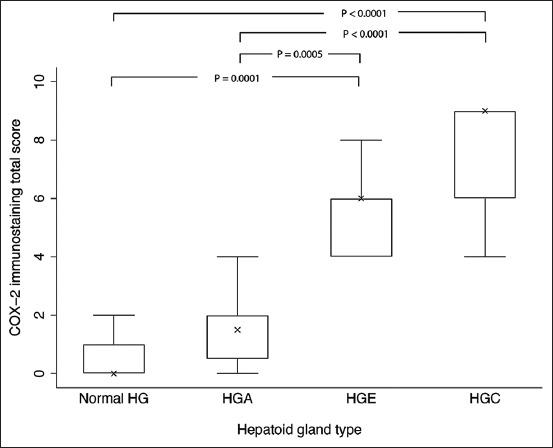
The cyclooxygenase-2 immunohistochemical score (IHS) in normal hepatoid gland tissues and three types of canine hepatoid gland neoplasms. The IHS of hepatoid gland carcinomas exhibited significantly higher than that in the normal hepatoid glands and hepatoid gland adenomas (p < 0.0001).

The histopathological findings of normal and neoplastic hepatoid glands are demonstrated in Figures-1a, c, e, and g. Regarding the tumor characteristic, there were 20 HGAs, 16 HGEs, and 15 HGCs. The immunohistochemical analysis of COX-2 revealed that the staining pattern was predominantly localized in the cytoplasm and occasional perinuclear region of neoplastic cells, while there was no staining of the surrounding stroma. The result of COX-2 immunoexpression in normal and neoplastic hepatoid glands is shown in Table-3. There were significant differences in COX-2 expression among normal and HGNs. Out of 10 normal hepatoid glands, there were 6/10 (60%) exhibit immunonegative (Figure-1b). In HGAs samples, 15/20 (75%) were weak COX-2 expression, characterized by faint cytoplasmic staining of the mature hepatoid cells, but negative in basaloid reserved cells (Figure-1d). As for HGEs, weak to moderate COX-2 expression was detected and is shown in Figure-1f. In HGCs cases, strong COX-2 expression was observed in 8/15 (53.3%), in which 3/8 (37.5%) showed anaplastic features with intense positive immunolabeling (Figure-1h). The differences in IHS of COX-2 among the normal and neoplastic hepatoid glands are elaborated in Figure-2. The IHS of HGCs exhibited significantly higher than the score of normal hepatoid glands and HGAs (p < 0.0001). The IHS of normal hepatoid glands and HGAs were not significantly different.

**Table-3:** The COX-2 expression in normal and neoplastic hepatoid glands.

Histology diagnosis	Number of samples	COX-2 expression				p-value

		Negative (%)	Weak (%)	Moderate (%)	Strong (%)	
Normal	10	6 (60.0)	4 (40.0)	0 (0)	0 (0)	<0.001
HGA	20	5 (25.0)	15 (75.0)	0 (0)	0 (0)	
HGE	16	0 (0)	7 (43.7)	9 (56.3)	0 (0)	
HGC	15	0 (0)	2 (13.3)	5 (33.3)	8 (53.3)	

HGA=Hepatoid gland adenomas, HGEs=Hepatoid gland epitheliomas, HGC=Hepatoid gland carcinoma, COX-2=Cyclooxygenase-2

### Association of clinicopathological features with the type of HGNs

The association of clinicopathological features and type of HGNs are summarized in Table-2. There were significant differences between type of HGNs and various clinicopathologic characters, including sex (p = 0.009), tumor size (p < 0.001), skin ulceration (p = 0.038), tissue necrosis (p < 0.001), tissue invasion (p < 0.001), and lymphatic metastasis (p = 0.001).

### Association of clinicopathological features with COX-2 expression in HGNs

The associations between clinicopathological variables and COX-2 expression in HGAs and HGEs are shown in Tables-4 and 5, respectively. The statistical evaluation of COX-2 expression according to age, sex, tumor size and location, skin ulceration and necrosis, tissue invasion, and lymphatic metastasis revealed no significant association in both HGAs and HGEs. On the other hand, COX-2 expression in HGCs was statistical significantly associated with the presence of tissue necrosis (p = 0.029) and tissue invasion (p = 0.007) (Table-6). Most of the strong COX-2 expressions were observed in tumors with massive necrosis (53.3%) and tumors with invasion to the surrounding tissues (80%). Strong intracytoplasmic immunolabeling was detected in 6/7 (85.7%) of HGC cases with lymphatic metastasis.

**Table-4:** Associations of clinicopathological features with COX-2 expression in HGAs.

Clinicopathologic features	Number of samples	COX-2 expression				p-value

		Negative (%)	Weak (%)	Moderate (%)	Strong (%)	
Age, years						0.613
<10	14	3 (21.4)	11 (78.6)	0 (0.0)	0 (0.0)	
>10	6	2 (33.3)	4 (66.7)	0 (0.0)	0 (0.0)	
Sex						1.000
Intact females	1	0 (0.0)	1 (100.0)	0 (0.0)	0 (0.0)	
Spayed females	2	0 (0.0)	2 (100.0)	0 (0.0)	0 (0.0)	
Castrated males	0	0 (0.0)	0 (0.0)	0 (0.0)	0 (0.0)	
Intact males	17	5 (29.4)	12 (70.6)	0 (0.0)	0 (0.0)	
Tumor size, cm						1.000
<3	12	3 (25.0)	9 (75.0)	0 (0.0)	0 (0.0)	
3–5	8	2 (25.0)	6 (75.0)	0 (0.0)	0 (0.0)	
>5	0	0 (0.0)	0 (0.0)	0 (0.0)	0 (0.0)	
Location						0.053
Perianal	17	3 (17.6)	14 (82.4)	0 (0.0)	0 (0.0)	
Tail based	2	2 (100.0)	0 (0.0)	0 (0.0)	0 (0.0)	
Prepuce	1	0 (0.0)	1 (100.0)	0 (0.0)	0 (0.0)	
Perivulva	0	0 (0.0)	0 (0.0)	0 (0.0)	0 (0.0)	
Ulceration						0.560
Present	5	2 (40.0)	3 (60.0)	0 (0.0)	0 (0.0)	
Absent	15	3 (20.0)	12 (80.0)	0 (0.0)	0 (0.0)	
Necrosis						1.000
Present	2	0 (0.0)	2 (100.0)	0 (0.0)	0 (0.0)	
Absent	18	5 (27.8)	13 (72.2)	0 (0.0)	0 (0.0)	
Tissue invasion						N/A
Present	0	0 (0.0)	0 (0.0)	0 (0.0)	0 (0.0)	
Absent	20	5 (25.0)	15 (75.0)	0 (0.0)	0 (0.0)	
Lymphatic metastasis						0.140
Present	0	0 (0.0)	0 (0.0)	0 (0.0)	0 (0.0)	
Absent	17	3 (17.6)	14 (82.4)	0 (0.0)	0 (0.0)	
N/A	3	2 (66.7)	1 (33.3)	0 (0.0)	0 (0.0)	

HGAs=Hepatoid gland adenomas, COX-2=Cyclooxygenase-2

**Table-5:** Associations of clinicopathological features with COX-2 expression in HGEs.

Clinicopathologic features	Number of samples	COX-2 expression				p-value

		Negative (%)	Weak (%)	Moderate (%)	Strong (%)	
Age, years						1.000
<10	7	0 (0.0)	3 (42.9)	4 (57.1)	0 (0.0)	
>10	9	0 (0.0)	4 (44.4)	5 (55.6)	0 (0.0)	
Sex						1.000
Intact females	1	0 (0.0)	0 (0.0)	1 (100.0)	0 (0.0)	
Spayed females	1	0 (0.0)	0 (0.0)	1 (100.0)	0 (0.0)	
Castrated males	5	0 (0.0)	3 (60.0)	2 (40.0)	0 (0.0)	
Intact males	9	0 (0.0)	4 (44.4)	4 (55.6)	0 (0.0)	
Tumor size, cm						0.106
<3	5	0 (0.0)	4 (80.0)	1 (20.0)	0 (0.0)	
3–5	10	0 (0.0)	3 (30.0)	7 (70.0)	0 (0.0)	
>5	1	0 (0.0)	0 (0.0)	1 (100.0)	0 (0.0)	
Location						0.437
Perianal	15	0 (0.0)	6 (40.0)	9 (60.0)	0 (0.0)	
Tail based	1	0 (0.0)	1 (100.0)	0 (0.0)	0 (0.0)	
Prepuce	0	0 (0.0)	0 (0.0)	0 (0.0)	0 (0.0)	
Perivulva	0	0 (0.0)	0 (0.0)	0 (0.0)	0 (0.0)	
Ulceration						1.000
Present	9	0 (0.0)	4 (44.4)	5 (55.6)	0 (0.0)	
Absent	7	0 (0.0)	3 (42.9)	4 (57.1)	0 (0.0)	
Necrosis						0.060
Present	7	0 (0.0)	1 (14.3)	6 (85.7)	0 (0.0)	
Absent	9	0 (0.0)	6 (66.7)	3 (33.3)	0 (0.0)	
Tissue invasion						N/A
Present	0	0 (0.0)	0 (0.0)	0 (0.0)	0 (0.0)	
Absent	16	0 (0.0)	7 (43.8)	9 (56.2)	0 (0.0)	
Lymphatic						1.000
Metastasis	0	0 (0.0)	0 (0.0)	0 (0.0)	0 (0.0)	
Present	13	0 (0.0)	6 (46.2)	7 (53.8)	0 (0.0)	
Absent	3	0 (0.0)	1 (33.3)	2 (66.7)	0 (0.0)	
N/A						

HGEs=Hepatoid gland epithelioma, COX-2=Cyclooxygenase-2

**Table-6:** Associations of clinicopathological features with COX-2 expression in HGCs.

Clinicopathologic features	Number of samples	COX-2 expression				p-value

		Negative (%)	Weak (%)	Moderate (%)	Strong (%)	
Age, years						1.000
<10	5	0 (0.0)	1 (20.0)	2 (40.0)	2 (40.0)	
>10	10	0 (0.0)	1 (10.0)	3 (30.0)	6 (60.0)	
Sex						0.920
Intact females	0	0 (0.0)	0 (0.0)	0 (0.0)	0 (0.0)	
Spayed females	3	0 (0.0)	0 (0.0)	2 (66.7)	1 (33.3)	
Castrated males	6	0 (0.0)	1 (16.7)	2 (33.3)	3 (50.0)	
Intact males	6	0 (0.0)	1 (16.7)	1 (16.7)	4 (66.6)	
Tumor size, cm						0.055
<3	0	0 (0.0)	0 (0.0)	0 (0.0)	0 (0.0)	
3–5	6	0 (0.0)	2 (33.3)	3 (50.0)	1 (16.7)	
>5	9	0 (0.0)	0 (0.0)	2 (22.2)	7 (77.8)	
Location						0.888
Perianal	9	0 (0.0)	1 (11.1)	3 (33.3)	5 (55.6)	
Tail based	4	0 (0.0)	1 (25.0)	1 (25.0)	2 (50.0)	
Prepuce	1	0 (0.0)	0 (0.0)	0 (0.0)	1 (100.0)	
Perivulva	1	0 (0.0)	0 (0.0)	1 (100.0)	0 (0.0)	
Ulceration						0.177
Present	10	0 (0.0)	1 (10.0)	5 (50.0)	4 (40.0)	
Absent	5	0 (0.0)	1 (50.0)	0 (0.0)	4 (80.0)	
Necrosis						0.029
Present	12	0 (0.0)	0 (0.0)	5 (41.7)	7 (53.3)	
Absent	3	0 (0.0)	2 (66.7)	0 (0.0)	1 (33.3)	
Tissue invasion						0.007
Present	10	0 (0.0)	1 (10.0)	1 (10.0)	8 (80.0)	
Absent	5	0 (0.0)	1 (20.0)	4 (80.0)	0 (0.0)	
Lymphatic						0.103
Metastasis	7	0 (0.0)	0 (0.0)	1 (14.3)	6 (85.7)	
Present	7	0 (0.0)	2 (28.6)	2 (42.8)	2 (28.6)	
Absent	1	0 (0.0)	0 (0.0)	1 (100.0)	0 (0.0)	
N/A						

HGCs=Hepatoid gland carcinomas, COX-2=Cyclooxygenase-2

## Discussion

Canine HGNs are common neoplasms arising from modified sebaceous glands and clinically characterized by rapid growth [38]. These neoplasms require early detection and definitive histopathological diagnosis for optimal treatment selection and prognosis. In this study, the type of HGNs was statistical significantly associated with various clinicopathologic characteristics, including sex, tumor size, tissue necrosis, skin ulceration, tissue invasion, and lymphatic metastasis. In the previous studies, benign hepatoid gland tumors have significantly high androgen, and low estrogen expressions [39, 40]. These tumors successfully regressed following castration or estrogen therapy [2]. However, in our study, HGAs occurred primarily in intact male dogs (53.1%), but in castrated male dogs, HGCs were predominant. Hepatoid gland carcinomas were also the highest neoplasm in spayed females (50%) (Table-3). Therefore, it is unclear whether sex hormones were truly associated with the malignancy of hepatoid gland tumors.

In our study, dogs with small (<3 cm) to medium (3–5 cm) tumor size were clinically speculated as benign (70.6%) and low-grade malignancy (41.7%) of canine HGNs, respectively. On the other hand, dogs with large tumor size (>5 cm) were diagnosed as HGCs (90%), similar to that in the previous studies [5, 41, 42]. The primary tumor size of over 5 cm of canine perianal adenocarcinomas with underlying tissue invasion revealed a high metastatic character. In addition, dogs with large tumor size had 4.5 and 11 times higher risk of recurrence and tumor-related mortality, respectively [5]. In this study, HGCs were associated with malignancy features, such as skin ulceration (66.7%), tissue necrosis (80%), invasion of surrounding tissues (66.7%), and lymphatic metastasis (46.7%). These results confirmed the findings of previous studies regarding the importance of these clinical characteristics for HGCs [5, 41].

This study was the first to document the immunohistochemical expression of COX-2 in normal and neoplastic canine hepatoid gland tissues along with clinicopathological features. The IHS of COX-2 was significantly different among the normal hepatoid glands, HGEs, and HGCs (p = 0.0001, p < 0.0001). The IHS of HGCs exhibited significantly higher than the score of normal hepatoid glands and HGAs (p < 0.0001). The study of Renkonen *et al*. [43] revealed that the immunoexpression of COX-2 protein was the lowest in normal oral mucosa and the intensity was gradually increased from hyperplasia to dysplasia and highest in invasive squamous cell carcinomas, which were consistent with our findings. Therefore, we suggest that an increased level of COX-2 immunostaining intensity may be associated with the levels of intracellular COX-2 protein and COX-2 mRNA. This hypothesis is supported by the study of Chan *et al*. [44] that the levels of COX-2 mRNA quantified by reverse transcription-PCR were increased 150-fold in the head and neck squamous cell carcinoma compared with normal oral mucosa. From our study, the strong COX-2 immunoexpression was mostly detected in malignant HGNs (53.3%). We speculate that these results were due to the mechanism mediating COX-2 and its role in tumorigenesis. Several studies have supported our finding that COX-2/PGE_2_ signaling pathways promote the processes of malignant transformation and tumor progression by affecting cell proliferation, cell adhesion, angiogenesis, tissue invasion, lymphatic metastasis, cell apoptosis, and immune-surveillance [12, 27]. Interestingly, three HGC cases with the presence of anaplastic features showed intense cytoplasmic immunolabeling. These results seemed attributable to the prominent Golgi apparatus in anaplastic cells. Theoretically, the Golgi apparatus is a key structure for transporting and secreting some essential enzymes which are overexpressed in cancer cells, such as COX-2 [45, 46]. Heller *et al*. [25] reported that anaplastic carcinomas had a significantly higher COX-2 staining distribution, intensity, and index, compared with those for mammary adenocarcinomas (p < 0.0001).

In the present study, we demonstrate that there was no association between clinicopathological variables and COX-2 expression in both HGAs and HGEs. Conversely, strong COX-2 immunoexpression was significantly associated with tissue invasion and necrosis in HGCs. This might reflect the ability of COX-2 to promote tumor invasiveness by inducing the production and activation of membrane-type matrix metalloproteinases or by stimulating angiogenesis [47]. Our result was similar to the finding in inflammatory canine mammary carcinoma, in which Queiroga *et al*. [26] found that higher levels of COX-2 expression were significantly associated with tumors with rapid growth, tumors adhered to the skin, and tumors with invasion of underlying tissues. As for tissue necrosis, intense COX-2 expression was found in the necrotic area, where the inflammatory cells, including neutrophils, plasma cells, lymphocytes, and macrophages were prominent. This finding might suggest that COX-2 expression was regulated by inflammatory mediators, especially interleukin-1 (IL-1). It was well-documented that IL-1 increased the expression of COX-2 and subsequently increased the concentration of PGE_2_ as part of the pro-oncogenic cascade [17, 48]. In addition, the potential elucidation includes that COX-2 expression had developed to protect the cancer cells from apoptosis under hypoxic conditions [49]. Several studies have revealed that strong COX-2 staining was observed adjacent to the necrotic area, which also has been noted in reports of human gliomas, hepatocellular carcinoma, cervical cancer, and canine osteosarcoma [50–53]. According to the metastasis, we found that the expression of COX-2 was strong in HGC dogs with lymphatic metastasis (85.7%). The previous studies have supported our finding that the intense COX-2 immunolabeling was associated with lymph node metastasis, the development of distant metastasis, and a worse prognosis in canine mammary carcinomas [26, 27]. It is possible that the over-expression of COX-2 stimulated vascular endothelial growth factor-C upregulation and consequently promoted lymphangiogenesis which was the first step for tumor cells spreading to the regional lymph nodes [54, 55].

According to our findings, the high COX-2 expression in malignant HGNs is in accordance with several human and animal cancers [14, 15, 21, 24]. Together with the predominant of COX-2 immunolabeling in HGCs was significantly associated with tissue invasion and necrosis. Moreover, the intense COX-2 expression was mainly found in lymphatic metastatic cases. Thus, it seems plausible that COX-2 may have a role in this tumor progression and that COX-2 inhibitors may be useful as additional treatment in dogs with HGCs. However, the exact role of COX-2 in canine hepatoid gland tumorigenesis is still unclear, and it needs to be more clearly elucidated. In both human and animal studies, they documented that COX-2 expression was associated with disease progression and poor clinical outcomes in various cancers, determining the role of COX-2 expression in prognostic values, such as disease-free interval and survival rate [24, 26, 56]. In addition, there had been a great interest in anti-COX-2 therapies, which showed anti-neoplastic effects, including inhibition of cell cycle arrest, angiogenesis, and increase of the immune response at the tumor site [28, 57]. Whether, the use of COX-2 inhibitors plays a role in the expression of COX-2 receptor in the tissue sections or not remains unclear. Unfortunately, access to information on previously used nonsteroidal anti-inflammatory drugs, especially COX-2 inhibitors, as well as therapeutic voiding times was limited in this study. Therefore, our further studies should investigate COX-2 expression as a prognostic indicator and the therapeutic potential of COX-2 inhibitors as an additional treatment for canine HGCs. These upcoming findings will be important in cancer treatment and prognosis.

## Conclusion

Our study demonstrated for the 1^st^ time that the IHS of COX-2 was significantly highest in canine HGCs compared with normal hepatoid glands, HGAs and HGEs. In addition, the strong COX-2 expression was associated with some clinicopathological features of tumor progression in HGCs. Therefore, these preliminary results suggest that COX-2 may be involved in tumorigenesis and may have potential in treatment decisions. Prospective studies are needed to assess the role of COX-2 as a prognostic factor as well as the COX-2 inhibitors as a novel targeted therapy for canine HGCs.

## Authors’ Contributions

HB and PC: Contributed to the study conception and design. HB and SC: Performed the experiments. HB and PC: Contributed to sample collection and preparation. HB, TM, and SC: Analyzed immunohistochemistry data. ST: Performed the statistical analysis. PC, HB, TM, and ST: Drafted and revised the manuscript. All authors have read and approved the final manuscript.
